# Supplementary material for: The roles of history, chance, and natural selection in the evolution of antibiotic resistance
Source: eLife. 2021 Aug 25;10:e70676. doi: 10.7554/eLife.70676 (PMC8412936; doi:10.7554/eLife.70676)
Supplement: Figure 1—source data 1. [file elife-70676-fig1-data1.docx]

|  | **CEFTAZIDIME** | | | |  |  | **IMIPENEM** | | | |
| --- | --- | --- | --- | --- | --- | --- | --- | --- | --- | --- |
|  | **0.5X** | **1X** | **2X** | **4X** |  |  | **0.5X** | **1X** | **2X** | **4X** |
| **B1** | 2.65 | 5.3 | 10.6 | 21.2 |  | **B1** | 0.03125 | 0.0625 | 0.125 | 0.25 |
| **B2** | 1.33 | 2.67 | 5.34 | 10.68 |  | **B2** | 0.052 | 0.104 | 0.208 | 0.416 |
| **B3** | 2 | 4 | 8 | 16 |  | **B3** | 0.03125 | 0.0625 | 0.125 | 0.25 |
| **P1** | 8 | 16 | 32 | 64 |  | **P1** | 0.02605 | 0.0521 | 0.1042 | 0.2084 |
| **P2** | 5.33 | 10.66 | 21.32 | 42.64 |  | **P2** | 0.03125 | 0.0625 | 0.125 | 0.25 |
| **P3** | 2.65 | 5.3 | 10.6 | 21.2 |  | **P3** | 0.02605 | 0.0521 | 0.1042 | 0.2084 |

**Figure 1-source data 1. Concentrations of CAZ and IMI (mg/L) added to the broth at different intervals of the evolution experiments**
