# Supplementary material for: The roles of history, chance, and natural selection in the evolution of antibiotic resistance
Source: eLife. 2021 Aug 25;10:e70676. doi: 10.7554/eLife.70676 (PMC8412936; doi:10.7554/eLife.70676)
Supplement: Figure 4—source data 1. — The average resistance levels (mg/L) and SEM are shown in the table. Replicates highlighted acquired the same mutation. [file elife-70676-fig4-data1.docx]

| Selection phase in CAZ | | | | | | | | | |
| --- | --- | --- | --- | --- | --- | --- | --- | --- | --- |
|  | Driver mutation in *adeJ* | CAZ (d3) | | CAZ (d12) | | IMI (d12) | | CIP (d12) | |
| B1_1 | I383F | 21.14 ± | 0.00 | 42.40 ± | 0.00 | 0.33 ± | 0.00 | 0.50 ± | 0.00 |
| B1_2 | *adeN* Δ3bp (541-543) | 21.14 ± | 0.00 | 42.40 ± | 0.00 | 0.12 ± | 0.02 | 0.13 ± | 0.00 |
| B1_3 | Q167R | 21.14 ± | 0.00 | 42.40 ± | 0.00 | 0.17 ± | 0.00 | 1.00 ± | 0.00 |
| B2_1 | Q176K | 2.64 ± | 0.00 | 42.40 ± | 0.00 | 0.17 ± | 0.00 | 0.06 ± | 0.00 |
| B2_2 | K15* | 5.29 ± | 0.00 | 31.80 ± | 6.12 | 0.17 ± | 0.00 | 0.13 ± | 0.00 |
| B2_3 | A290T | 7.93 ± | 1.53 | 42.40 ± | 0.00 | 0.25 ± | 0.05 | 0.13 ± | 0.00 |
| B3_1 | G288S | 21.14 ± | 0.00 | 42.40 ± | 0.00 | 0.17 ± | 0.00 | 0.13 ± | 0.00 |
| B3_2 | F136L | 31.72 ± | 6.10 | 21.20 ± | 0.00 | 0.17 ± | 0.00 | 0.50 ± | 0.00 |
| B3_3 | G288S | 42.29 ± | 0.00 | 42.40 ± | 0.00 | 0.33 ± | 0.00 | 0.13 ± | 0.00 |
| P1_1 | A1007S | 84.57 ± | 0.00 | 84.80 ± | 0.00 | 0.17 ± | 0.00 | 16.03 ± | 0.00 |
| P1_2 | T743P | 84.57 ± | 0.00 | 169.60 ± | 0.00 | 0.33 ± | 0.00 | 16.03 ± | 0.00 |
| P1_3 | Q176K | 84.57 ± | 0.00 | 127.20 ± | 24.48 | 0.17 ± | 0.00 | 8.02 ± | 0.00 |
| P2_1 | Q176R | 10.57 ± | 0.00 | 84.80 ± | 0.00 | 0.08 ± | 0.00 | 8.02 ± | 0.00 |
| P2_2 | *ftsI* Δ3bp (1072-1074) | 5.29 ± | 0.00 | 42.40 ± | 0.00 | 0.08 ± | 0.00 | 0.50 ± | 0.00 |
| P2_3 | Q176R | 7.93 ± | 1.53 | 42.40 ± | 0.00 | 0.17 ± | 0.00 | 8.02 ± | 0.00 |
| P3_1 | V158L (77%), F94L (28%) | 3.96 ± | 0.76 | 63.60 ± | 12.24 | 0.08 ± | 0.00 | 8.02 ± | 0.00 |
| P3_2 | V158L | 2.64 ± | 0.00 | 84.80 ± | 0.00 | 0.17 ± | 0.00 | 8.02 ± | 0.00 |
| P3_3 | F136S | 3.96 ± | 0.76 | 63.60 ± | 12.24 | 0.08 ± | 0.00 | 8.02 ± | 0.00 |
| Selection phase in IMI | | | | | | | | | |
|  | Driver mutation in *ftsI* | IMI (d3) | | IMI (d12) | | CAZ (d12) | | CIP (d12) | |
| B1_1 | A579V | 0.17 ± | 0.00 | 0.33 ± | 0.00 | 4 ± | 0 | 0.42 ± | 0.07 |
| B1_2 | T506I | 0.14 ± | 0.02 | 0.33 ± | 0.00 | 5.33 ± | 1.09 | 0.50 ± | 0.00 |
| B1_3 | T506I | 0.14 ± | 0.02 | 0.33 ± | 0.00 | 8.00 ± | 0.00 | 0.42 ± | 0.07 |
| B2_1 | A579V | 0.17 ± | 0.00 | 0.33 ± | 0.00 | 4.00 ± | 0.00 | 0.50 ± | 0.00 |
| B2_2 | G524C | 0.22 ± | 0.05 | 0.33 ± | 0.00 | 2.00 ± | 0.00 | 0.42 ± | 0.07 |
| B2_3 | A579T | 0.17 ± | 0.00 | 0.33 ± | 0.00 | 4.00 ± | 0.00 | 0.10 ± | 0.02 |
| B3_1 | A583V | 0.17 ± | 0.00 | 0.17 ± | 0.00 | 4.00 ± | 0.00 | 0.13 ± | 0.00 |
| B3_2 | G574S | 0.08 ± | 0.00 | 0.17 ± | 0.00 | 2.00 ± | 0.00 | 0.08 ± | 0.02 |
| B3_3 | G524S | 0.17 ± | 0.00 | 0.66 ± | 0.00 | 2.00 ± | 0.00 | 0.06 ± | 0.00 |
| P1_1 | H530Y | 0.33 ± | 0.00 | 0.33 ± | 0.00 | 16.00 ± | 0.00 | 16.03 ± | 0.00 |
| P1_2 | A583D | 0.17 ± | 0.00 | 0.33 ± | 0.00 | 10.67 ± | 2.18 | 10.69 ± | 2.18 |
| P1_3 | A579V | 0.17 ± | 0.00 | 0.66 ± | 0.00 | 5.33 ± | 1.09 | 16.03 ± | 0.00 |
| P2_1 | S395V | 0.17 ± | 0.00 | 1.33 ± | 0.00 | 16.00 ± | 0.00 | 2.67 ± | 1.09 |
| P2_2 | A579V | 0.17 ± | 0.00 | 1.33 ± | 0.00 | 53.33 ± | 8.71 | 4.01 ± | 0.00 |
| P2_3 | P580A | 0.17 ± | 0.00 | 0.66 ± | 0.00 | 10.67 ± | 2.18 | 5.34 ± | 1.09 |
| P3_1 | S539P | 0.17 ± | 0.00 | 1.33 ± | 0.00 | 8.00 ± | 0.00 | 4.01 ± | 0.00 |
| P3_2 | A578T | 0.17 ± | 0.00 | 0.66 ± | 0.00 | 4.00 ± | 0.00 | 8.02 ± | 0.00 |
| P3_3 | A579V | 0.17 ± | 0.00 | 0.66 ± | 0.00 | 2.00 ± | 0.00 | 8.02 ± | 0.00 |

**Figure 4-source data 1. Putative driver mutations and resistance levels of the replicate populations after 12 days evolving in presence of CAZ or IMI.** The average resistance levels (mg/L) and SEM are shown in the table. Replicates highlighted acquired the same mutation.
